# Modulation of Telomeres in Alternative Lengthening of Telomeres Type I Like Human Cells by the Expression of Werner Protein and Telomerase

**DOI:** 10.1155/2012/806382

**Published:** 2012-03-28

**Authors:** Aisha Siddiqa, David Cavazos, Jeffery Chavez, Linda Long, Robert A. Marciniak

**Affiliations:** ^1^Department of Medicine, University of Texas Health Science Center, 7703 Floyd Curl Drive, San Antonio, TX 78229-3900, USA; ^2^Department of Nutritional Sciences, School of Human Ecology, The University of Texas at Austin, Austin, TX 78712, USA; ^3^Department of Cellular and Structural Biology, University of Texas Health Science Center, 7703 Floyd Curl Drive, San Antonio, TX 78229-3900, USA; ^4^South Texas Veterans Healthcare Administration, San Antonio, TX 78229, USA

## Abstract

The alternative lengthening of telomeres (ALT) is a recombination-based mechanism of telomere maintenance activated in 5–20% of human cancers. In *Saccharomyces cerevisiae*, survivors that arise after inactivation of telomerase can be classified as type I or type II ALT. In type I, telomeres have a tandem array structure, with each subunit consisting of a subtelomeric Y′ element and short telomere sequence. Telomeres in type II have only long telomere repeats and require Sgs1, the *S. cerevisiae* RecQ family helicase. We previously described the first human ALT cell line, AG11395, that has a telomere structure similar to type I ALT yeast cells. This cell line lacks the activity of the Werner syndrome protein, a human RecQ helicase. The telomeres in this cell line consist of tandem repeats containing SV40 DNA, including the origin of replication, and telomere sequence. We investigated the role of the SV40 origin of replication and the effects of Werner protein and telomerase on telomere structure and maintenance in AG11395 cells. We report that the expression of Werner protein facilitates the transition in human cells of ALT type I like telomeres to type II like telomeres in some aspects. These findings have implications for the diagnosis and treatment of cancer.

## 1. Introduction

As progressive loss of telomere DNA is associated with senescence [1], maintenance of telomere function is essential for indefinite cell proliferation. Most cancer cells rely on expression of telomerase for suppression of telomere shortening [2]. However 5%–20% percent of cancers maintain telomeres by the alternative lengthening of telomere (ALT), a recombination-based mechanism [3].

Telomere maintenance mechanisms are a potential prognostic indicator [3] and promising target in cancer diagnosis and therapy [4–6]. Increasing evidence supports that Werner protein (WRN), a RecQ helicase and exonuclease, plays a direct role in telomere maintenance [7] and promotion of tumor cell growth [8]. WRN epigenetic silencing in human cancers leads to hypersensitivity to treatment with a number of chemotherapeutic drugs [9]. Germline mutations in the WRN gene cause an autosomal recessive disorder, Werner syndrome (WS). WS is characterized by symptoms suggestive of premature aging and by the development of mesenchymal neoplasms [10]. Strikingly, the ALT mechanism is more prevalent in tumors arising from tissues of mesenchymal origin, such as osteosarcomas, than in those of epithelial origin [11]. It has been suggested that the telomere-telomere recombination in WRN-deficient, telomere dysfunctional cells promotes escape from senescence and engagement of the ALT pathway [12]. Werner protein also colocalizes with telomeres in human ALT cells [13].

*S. cerevisiae* cells that lack functional telomerase undergo telomere attrition and lose viability [14]. Rare cells escape senescence and two types of survivors arise. Type I ALT survivors have telomeres that have a tandem array structure. The repeat unit in the array consists of a subtelomeric Y′ element containing an ARS (yeast origin of replication) associated with short telomeric TG_1−3_ repeats. This repeat unit is amplified as a tandem array structure at chromosome termini. Type II survivors have little or no amplification of Y′ elements, but instead have long, heterogeneous, TG_1−3_ repeats extending up to several kilobase pairs (kbp) [15, 16]. The generation of type I cells depends on expression of proteins involved in recombination, including RAD52 and RAD51. Type II cells depend on expression of Sgs1, the *S. cerevisiae* RecQ family helicase, in addition to recombination proteins RAD52 and RAD50 [13, 17]. WRN can complement Sgs1 deficiency in type II ALT cells of *S. cerevisiae* [17]. *Sgs1* deletion also facilitates the generation of survivors that grow independent of Rad52. Although *tlc1rad52 *yeast does not form survivors of telomere dysfunction, *tlc1rad52sgs1* triple mutants readily generated survivors [18].

Nearly all of the human ALT cell lines analyzed to date have characteristics similar to that of *S. cerevisiae* type II ALT. Most human ALT cells have long and heterogeneous telomeres, ranging from 2 to 20 kb within an individual cell and have ALT-associated promyelocytic leukemia bodies (APBs) [19]. APBs contain the constitutive components of promyelocytic leukemia bodies, telomere DNA, and the proteins involved in DNA replication and recombination including RAD51, RAD52, RAD50, and WRN [3].

One immortalized human cell line has a “tandem array” telomere structure similar to that of type I ALT in yeast [20, 21]. AG11395 is an SV40 T-antigen transformed, immortalized fibroblast cell line derived from an individual diagnosed with Werner syndrome [22]. It does not contain APBs and lacks the Werner syndrome protein. The chromosome termini of AG11395 consist of a repeat unit containing 2.5 kb of SV40 DNA and a variable amount of TTAGGG telomere sequence repeats. The SV40 DNA integrated into the telomere in this cell line contains the regulatory regions, which include the origin of replication and the early and late promoter sequences [21]. This cell line offers a unique system to investigate the role of the WRN protein and tandem array telomeres in human ALT telomere maintenance. Here we determine whether telomere maintenance in AG11395 involves a functioning SV40 origin of replication and we describe the effect on type I like structures of expression of WRN protein.

## 2. Materials and Methods

### 2.1. Cell Lines and Culture

AG11395, WI38 75.1, and GM847 were obtained from the Coriell Cell Repository (Camden, NJ). HeLa and COS cells were obtained from the American Type Culture Collection (Manassas, VA). Cells were grown in 5% CO_2_ under conditions recommended by the supplier.

### 2.2. Quantitative Polymerase Chain Reaction (qPCR)

To determine whether in AG11395 the SV40 origin of replication integrated at telomeres is functioning, we cotransfected pRLSV40 (Promega. Madison, WI) that has SV40 origin of replication and pRLCMV (Promega. Madison, WI) that does not contain an SV40 origin of replication in a 1 : 1 ratio in AG11395. The ratio of pRLSV40 to pRLCMV was assessed by qPCR, with primers specific to each plasmid using Express SYBR Green qPCR reagents (Invitrogen) in a Mx3000P thermal cycler (Stratagene, La Jolla, CA).

The primers used included

pRLSV40-forward primer: 5′-GGCTTTTTTGGAGGCCTAGG-3′
pRLSV40-reverse primer: 5′-CGAGACTGTTGTGTCAGAAGAATCA-3′
pRLCMV-forward primer: 5′-CGTGTACGGTGGGAGGTC-3′
pRLCMV-reverse primer: 5′-CAATAAAGCTTCTAGTGATCTGACG-3′
SV40 regulatory sequences before and after WRN expression were quantified by qPCR with the following primers:
5′-CCT CAG TAA GCA CAG CAA GCA T-3′
5′-AAT GGC CTG AGT GTG CAA AGA-3′

For internal control, the *β*-globin gene was amplified with

5′-TGA AGG CTC ATG GCA AGA AA-3′
5′-GGT GAG CCA GGC CAT CAC-3′

Amplification conditions for all qPCR were 10 minutes at 95°C, followed by 30 cycles of 30 seconds at 95°C followed by 60 seconds at 60°C.

### 2.3. Retroviral Infection

To produce infectious retrovirus, the phoenix packaging cell line (kindly provided by Dr. Garry P. Nolan, Stanford University) was transfected with pLXIN (Clontech, Mountain View, CA) and pLXIN/WRN using Fugene-6 (Roche Applied Sciences, Indianapolis, IN) per the manufacturer's instructions. Forty-eight hours after transfection, supernatant was filtered through a 0.45 *μ*m filter to obtain a cell-free stock of virus. The viral supernatant was added to AG11395 cells and incubated 24 hours. The infected cells were selected by addition of 200 *μ*g/mL G418 (Sigma, St. Louis, MO). To produce cell lines expressing telomerase reverse transcriptase, pBABE/hTERT and pBABE/hTERT HA (Robert Weinberg, M.I.T.) were used as described above with the exception that infected cells were selected by addition of 0.1 *μ*g/mL puromycin (Mediatech, Manassas, VA). The puromycin-selected telomerase expressing cells were tested as a mixed population. Given greater heterogeneity of expression in the WRN expressing clones, 3 individual subclones were isolated and selected for further study.

### 2.4. Western Blots

Ten micrograms of protein extract were resolved on an 8% SDS-PAGE gel and transferred to HyBond ECL membrane (New England Nuclear, Boston, MA). Affinity purified polyclonal rabbit anti-Werner and monoclonal mouse anti-actin antibodies (Abcam, Cambridge, MA) were used at a dilution of 1 : 1000. Western blotting was performed as described [23].

### 2.5. Indirect Immunofluorescence

Fixation and indirect immunofluorescence were done as described [24]. Antibodies and dilutions used were mouse monoclonal anti-PML (Santa Cruz Biotech, Santa Cruz, CA) at a dilution of 1 : 100, anti-TRF1 (hybridoma supernatant 4E4, GeneTex, San Antonio, TX), at a dilution of 1 : 5, and affinity-purified polyclonal rabbit anti-Werner antibody was used at a dilution of 1 : 100. Cy3-conjugated anti-mouse (Molecular Probes, Carlsbad, CA) and FITC-conjugated anti-rabbit (Jackson labs, Bar Harbor, ME) at a dilution of 1 : 300 were used to detect anti-TRF1 anti-PML and anti-Werner. Nuclei were counterstained with 4,6-diamidino-2-phenylindole dihydrochloride (DAPI) at a concentration of 100 ng/mL. Immunofluorescence images were obtained using a Nikon Eclipse TE200 microscopy system.

### 2.6. Telomerase Enzymatic Assays

Telomerase enzymatic activity was detected using the TRAPeze telomerase detection kit (Chemicon, Temecula, CA).

### 2.7. Quantification of Telomere Length by Quantitative Fluorescent *In Situ* Hybridization (Q-FISH)

Telomere lengths were quantified in arbitrary fluorescence units (AFUs) using a peptide nucleic acid (PNA) probe directly labeled with a Cy3 (Applied Biosystems, Foster City, CA) as previously described [25]. Cells were harvested by centrifugation at 200 × g. The pellet was resuspended in hypotonic KCl (0.067 M) for 5 min at 37°C and then fixed and washed in methanol/acetic acid (3 : 1). Q-FISH was performed as described by Poon and Lansdorp [25]. Briefly, fixed metaphase nuclei were dropped onto slides, hybridized with PNA probe, and images of telomeres and chromosomes were captured using digital fluorescence microscopy. The images were analyzed with the TFL-Telo program to detect the telomere-specific fluorescence signal. To compensate the inter-specimen hybridization fluctuation, plasmids containing 1.6 kb, and 800 bp telomere sequence were used as control. To check any variation in lamp or alignment of the optics, we measured the integrated fluorescence intensity (IFI) of 0.1 *μ*m beads for normalization between different experiments [25]. All experiments within a set in which quantitative length comparisons were made were dropped, hybridized, and analyzed in parallel at one time to minimize effects of probe integrity, hybridization efficiency or drift in the performance of the optical system.

### 2.8. Chromosome Orientation-FISH (CO-FISH)

Telomere sister chromatid exchange (T-SCE) was performed using chromosome-orientation-specific fluorescence *in situ* hybridization (CO-FISH). CO-FISH procedure was performed as described in [26]. Briefly, during S-phase, Bromodeoxyuridine (BrdU) and Bromodeoxycytidine (BrdC) nucleotides are incorporated into the newly synthesized DNA during one cell division. The BrdU-substituted DNA strands are degraded by Hoechst dye, UV, and exonuclease treatments. The parental G-rich and C-rich strands are visualized on the single-stranded sister chromatids by specific probes labeled with Cy3 (against the G-rich strand) or FITC (against the C-rich strand).

### 2.9. Combined IF/PNA FISH

Colocalization of PML protein with telomeres in WRN expressing AG11395 was confirmed by IF/FISH, with IF performed as described above. After incubation with secondary antibody, the immune complexes were cross-linked with 4% paraformaldehyde, dehydrated and processed for PNA FISH, as described for Q-FISH above.

## 3. Results

### 3.1. AG11395 Does Not Support the Replication of the SV40 Origin of Replication

**Figure 1:**
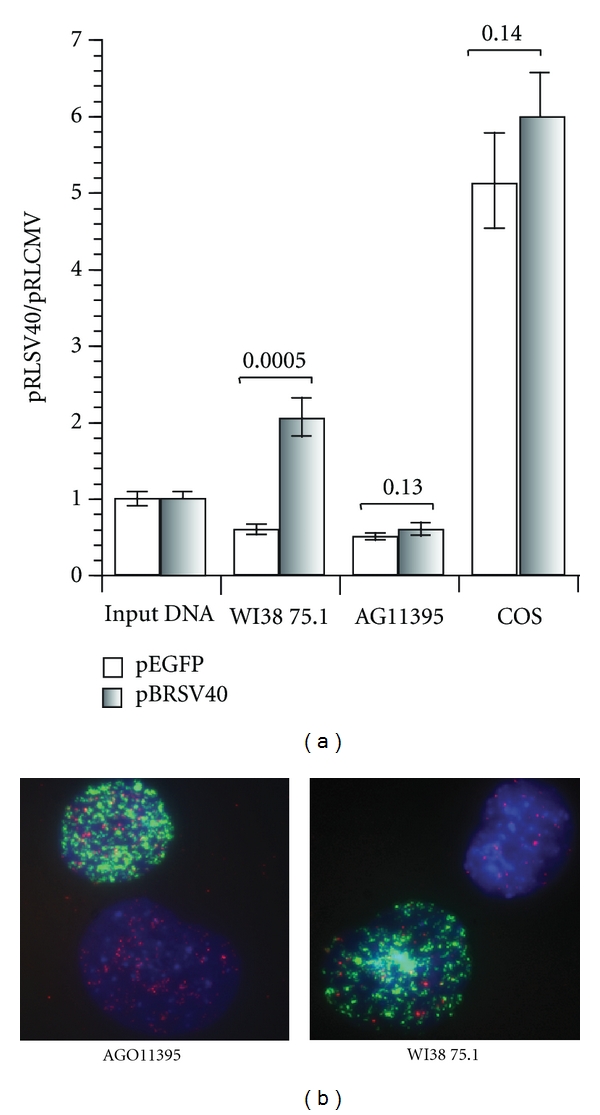
AG11395 does not support the replication of plasmids containing an SV40 origin of replication. (a) Three SV40-transformed cell lines were transfected with pRLSV40, which contains an SV40 origin of replication and pRLCMV, which does not contain an SV40 origin, in a 1 : 1 molar ratio along with pEGFP (as carrier DNA), or pBRSV40 (to supply wild-type T-antigen). Replication was measured by the ratio of pRLSV40 to pRLCMV as determined by qPCR, normalized to the ratio in the input DNA. AG11395 does not support replication of pRLSV40, even if wild-type T-antigen is provided by cotransfection. WI38 75.1 does support replication of pRLSV40, but only if wild-type T-antigen is provided by cotransfection. COS cells support efficient replication of pRLSV40, with or without additional T-antigen expression. (b) A short pulse (30 min) of EdU (5-ethynyl-2′-deoxyuridine) in AG11395 and WI38 75.1, S phase cell does not show a significant difference in the incorporation of EdU pattern in cells. Telomere pna FISH is (Cy3) red, EdU is (FITC) green, and DAPI-stained nuclei are blue.

The telomeres of AG11395 contain an SV40 sequence, which includes a wild-type sequence origin of replication [21]. One possible function of the nontelomere repeat sequence integrated into the telomeres of cell line AG11395 could be to provide a functional origin of replication integrated at telomeres. The Y′ element found integrated into the telomeres of type I survivors of telomere-induced senescence in *S. cerevisiae* contains a yeast autonomously replicating sequence (ARS) [27]. The ARSs in Y′ elements are nonfunctional in the proximity of telomere sequences [28, 29]. To assess whether any SV40 origin of replication is functional in cell line AG11395, we determined whether a plasmid containing a wild-type origin of replication would be replicated when transfected into this cell line (Figure 1). We co-transfected pRLSV40, which contains a functional SV40 origin of replication, and pRLCMV, which does not contain an SV40 origin of replication. Replication of the SV40 origin containing plasmid is measured as an increase in the ratio of pRLSV40 to pRLCMV DNA, as measured by qPCR. In addition, we tested whether inhibitors of replication of SV40 were present in the cell lines by additionally cotransfecting pBRSV40, which expresses wild-type SV40 T-antigen. As carrier DNA in those experiments was not including pBRSV40, plasmid pEGFP was used, allowing for visual monitoring of transfection efficiency (Figure 1(a)).

SV40 origin containing plasmid replication was assessed in 3 cell lines: AG11395, WI38 75.1 (an SV40-transformed, T-antigen expressing, ALT fibroblast cell line), and COS (which efficiently supports replication directed by the SV40 origin). Demonstrating that this methodology for measuring plasmid replication is functioning, we observed a substantial increase in the pRLSV40/pRLCMV ratio in COS cells, both with and without T-antigen provided in trans by transfection of plasmid pBRSV40 (Figure 1(a)). Neither AG11395 nor WI38 75.1 supported replication of the SV40 origin containing plasmid without expression of wild-type T antigen. The small decrease in the pRLSV40/pRLCMV measured in the cell lines not replicating the SV40 origin containing plasmid may reflect slight differences in the quality of the plasmid preparations used for transfection. WI38 75.1, but not AG11395, was capable of supporting the replication of the SV40 origin containing plasmid when T-antigen was provided by co-transfection of pBRSV40. These results show that SV40 origins of replications are not functional in AG11395 cells.

A short pulse (30 min) of EdU (5-ethynyl-2′-deoxyuridine) in S phase cell does not show a significant difference in the incorporation of EdU pattern in AG11395 and WI38 75.1 cells (Figure 1(b)), again confirming that SV40 origins in the telomeres of AG011395 cells are not functional.

### 3.2. Exogenously Expressed WRN Colocalizes with Telomeres in AG11395

**Figure 2:**
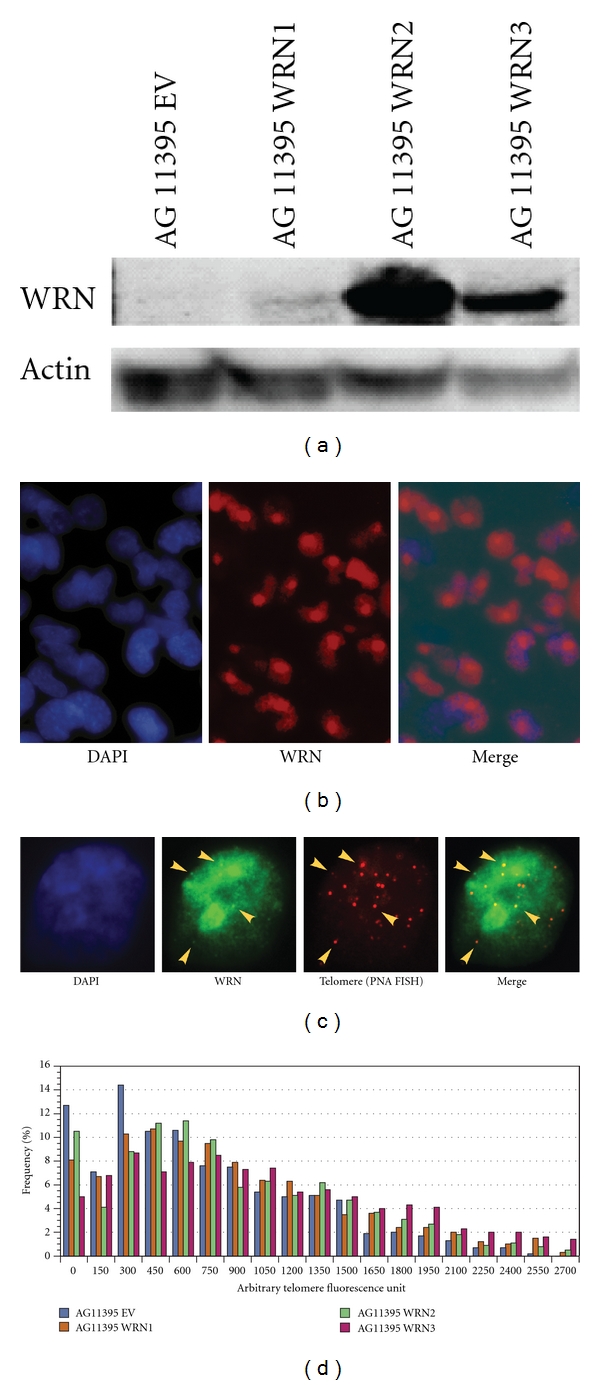
WRN expression in AG11395 cells. (a) Western blot of WRN expression in AG11395 clones. 10 ug of total protein was prepared from the empty vector-infected and WRN expressing cell lines and blotted with the antibodies indicated. (b) WRN transgene expression in AG11395 was analyzed by indirect immunofluorescence (IF). WRN is present at increased concentration in the nucleoli of AG11395 cells expressing the WRN transgene. (c) Exogenous WRN expressed in AG11395 colocalizes with telomeres. PNA FISH and IF were performed with antibodies to WRN and TRF1; nuclei were visualized with DAPI. (d) Effect of WRN expression on telomeres in AG11395 cells. Telomere length increases after WRN expression in AG11395 cells. Quantitation of telomere length was performed by PNA FISH in the three AG11395 empty-vector and three WRN expressing clones.

In *S. cerevisiae*, ALT type I survivors with a tandem array telomere structure initially arise, but grow poorly. With continued passage, these are replaced by type II survivors that have long, heterogeneous, TG_1–3_ repeats [16]. Survivors in *S. cerevisiae tlc1sgs1* cultures maintain a clear type I telomere structure and type II survivors do not arise, suggesting that *Sgs1* is important for the development of type II survivors. WRN facilitates telomere recombination and can complement *sgs1* deficiency in type II ALT cells of *S. cerevisiae* [30]. WRN also colocalizes with telomeres in human ALT type II cells [13]. Given these observations, we investigated the possible role of WRN in transition from a “type 1” like ALT telomere structure into a “type II” like ALT telomere structure in human cells. We cloned the full-length WRN coding sequence in retroviral vector pLXIN. AG11395 cells were infected with empty and WRN expressing pLXIN. Three WRN expressing clones (WRN1, WRN2, and WRN3) and three empty-vector clones (EV1, EV2, EV3) were selected for analysis. WRN expression was confirmed by western blot (Figure 2(a)) and indirect immunofluorescence (IF) (Figure 2(b)). We then determined the localization of WRN in AG11395. Indirect Immunostaining of WRN and TRF1 revealed colocalization of WRN with TRF1 (data not shown). We further confirmed WRN colocalization with telomere DNA by detecting the telomere with peptide nucleic acid fluorescent *in situ* hybridization (PNA FISH) combined with WRN IF (Figure 2(c)).

### 3.3. Sister Telomeres Loss (STL) Is Decreased in WRN Expressing AG11395

The localization of WRN at telomeres suggested that WRN might function in telomere metabolism in AG11395. Increase in STL has been reported in WRN-deficient fibroblasts and by the inhibition of WRN in HeLa cells [31]. To determine if WRN is functioning at telomeres when expressed in AG11395, we determined the frequency of STL in three control retrovirus-infected and three WRN expressing retrovirus-infected AG11395 subclones (Table 1). The expression of WRN in AG11395 decreased the rate of STLs from a mean of 10.4 per metaphase spread in the three empty vector-infected AG11395 cells to 7.1, 6.9 and 4.4 per metaphase spread in the AG11395 WRN1, WRN2, and WRN3 cells, respectively.

### 3.4. Expression of WRN Increases Telomere Repeat Sequences in AG11395

In yeast, Sgs1-dependent transition of type I ALT to type II ALT telomere structures is accompanied by appearance of long tracts of telomere repeats [17]. The presence of WRN at telomeres suggested that reexpression of WRN might similarly modulate the lengths of the telomere repeat tracts in AG11395. We quantified telomere repeats by quantitative PNA FISH in control and WRN-expressing clones (Figure 2(d)). We found that all three WRN expressing clones show increased telomere repeat signals at telomeres at population doubling (PD) 24 (Table 2). The WRN expressing clones AG11395 WRN1, WRN2, and WRN3 had mean telomere sequence lengths of 807, 771 and 919 AFUs, as compared to the mean telomere sequence lengths in empty vector-infected AG11395 cells EV1, EV2, and EV3 that measured 577, 547, and 449 AFUs, respectively.

### 3.5. SV40 Sequences Are Lost with Increasing Population Doubling (PD) after WRN Expression in AG11395

**Figure 3:**
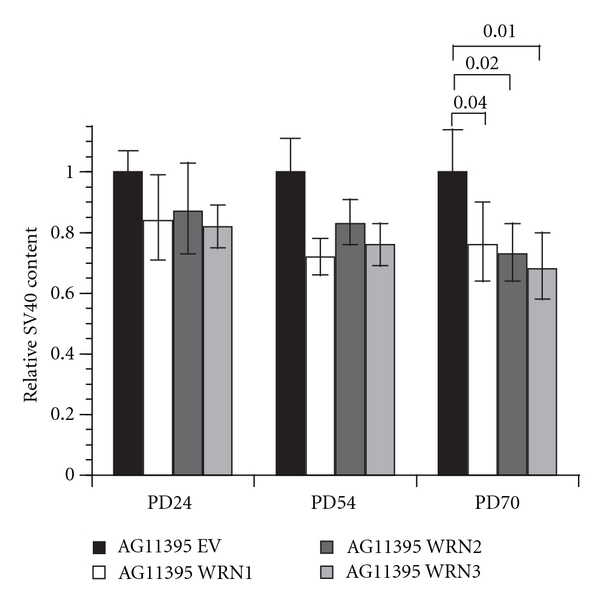
SV40 sequences decrease after WRN expression in AG11395 cells. (a) The SV40 DNA content in the WRN expressing AG11395 clones was determined by qPCR at the indicated population doublings and normalized to the SV40 DNA content of the empty vector infected clone at that population doubling. One-tailed probability values of *t*-tests are indicated.

In AG11395, the repetitive structure at telomeres contains both telomere and SV40 sequences [21]. In yeast, the transition from type I ALT to type II ALT telomere structures involves loss of a tandem array telomere structure and appearance of long tracts of telomere repeats [17]. After observing an increase in telomere sequence with expression of WRN protein in AG11395, we determined whether SV40 sequences were decreasing. We quantified the SV40 sequence by quantitative polymerase chain reaction (qPCR) at PD 24, 54, and 70 (Figure 3). All three WRN expressing clones showed a trend of decreasing SV40 sequence with increasing PDs when compared with the empty vector-infected clone at the same PD (Table 2). As analyzed by FISH, SV40 sequences detectable in AG11395 colocalize with telomere sequences [20]. The overall loss of SV40 sequences as measured by qPCR therefore indicates a loss of SV40 sequences from telomeres.

### 3.6. ALT Associated PML Bodies (APBs) Are Present in WRN Expressing AG11395 Cells

**Figure 4:**
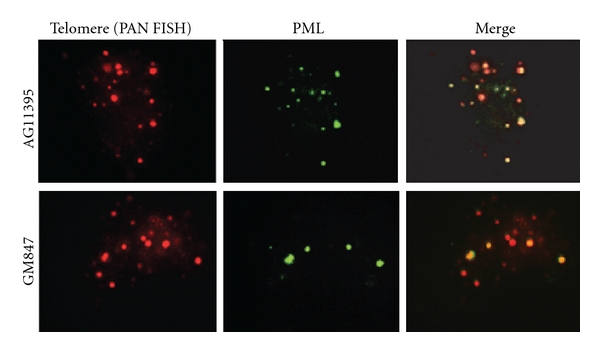
APBs can be detected after WRN expression in AG11395 cells. APBs can be detected by colocalization of PML antigen with telomere markers. Indirect immunofluorescence to detect the PML protein was combined with PNA FISH to detect telomere DNA sequences. PML indirect immunofluorescence (green) and telomere PNA FISH (red) show significant colocalization in the ALT cell line, GM847. After re-expression of WRN protein, similar colocalization is observed in a subset of cells in AG11395 WRN protein expressing subline.

APBs are characterized by the co-localization of the PML protein with telomere repeat binding factors in ALT cells and have been used as morphologic marker for the presence of ALT telomere maintenance [19]. The frequency of cells containing APBs is increased when cultures are enriched for cells in the G2/M phase of the cell cycle [32]. AG11395 cells do not contain APBs [20]. Instead, they have nuclear aggregates containing SV40 large T antigen and many of the same components as APBs. After WRN expression in AG11395, we observed few spots of TRF1 and PML colocalization in rare cells (data not shown). Following 1.5 ug/mL nocodazole arrest to enrich the G2/M phase cells we looked for APBs by combined IF/PNA FISH. After analyzing 300 cells, we found that 3% of cells show telomere and PML co-localization (Figure 4) and Table 2.

### 3.7. Expression of hTERT Restores Telomerase Activity in AG11395

**Figure 5:**
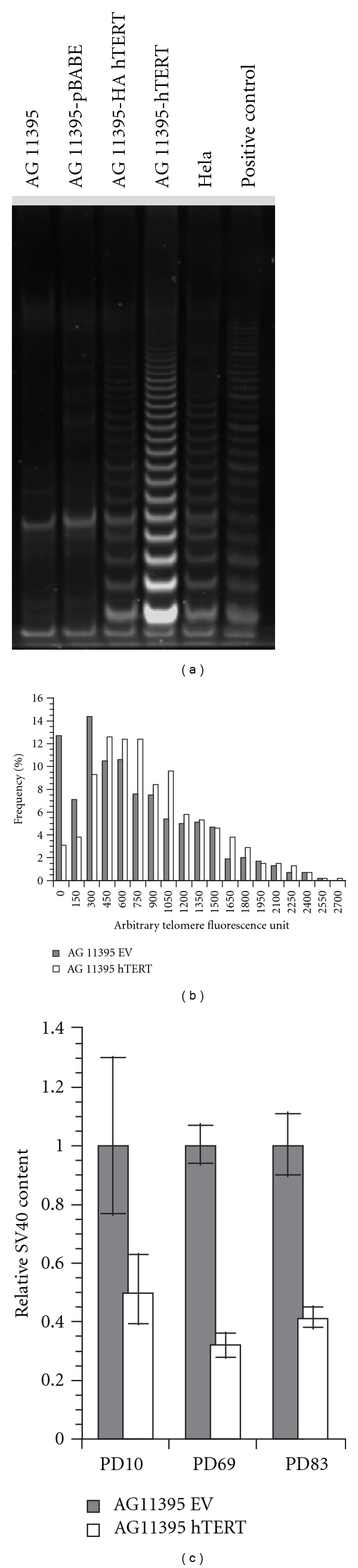
Expression of hTERT in AG11395 modulates telomere length. (a) TRAP assay on hTERT-expressing cell lines. Exogenous expression of hTERT makes AG11395 cells TRAP positive. (b) Q-FISH of telomere repeat content in empty vector and mixed telomerase expressing AG11395 cells. Expression of telomerase increased the median telomere repeat signal in the population and caused loss of the telomeres with minimal detectable telomere repeat signal. (c) Q-PCR for SV40 sequences in the empty vector and hTERT expressing AG11395 sublines. SV40 sequences rapidly decreased by more than 60% after hTERT expression in AG11395. With continued passage, further loss of SV40 sequences was not observed.

AG11395 cells lacked detectable telomerase activity as measured by the telomere repeat amplification protocol (TRAP) assay (Figure 5(a)) and lack expression of human telomerase reverse transcriptase (hTERT) mRNA by quantitative reverse-transcriptase PCR (data not shown). Telomerase activity can be reconstituted in some type II ALT cells by expression of hTERT [33]. To investigate if the ALT type I like cells can reconstitute human telomerase reverse transcriptase (hTERT) activity and alter telomere maintenance we produced AG11395 expressing pBABE-puro-hTERT [34]. Telomerase activity was assayed by TRAP [35] at PD10. AG11395 cells express telomerase and become TRAP positive (Figure 5(a)).

### 3.8. Expression of Telomerase in AG11395 Cells Lengthens Telomeres, Decreases STLs, and Induces Loss of SV40 Sequences

Expression of exogenous hTERT in ALT type II cells results in lengthening of the shortest telomeres [33]. WRN-deficient fibroblasts have higher frequency of short telomeres and STLs [31]. Telomerase preferentially acts on short telomeres in WS fibroblasts and thus reduces number of STLs [31]. Expression of telomerase in AG11395 cells resulted in lengthening of short telomeres (Figure 5(b)). As expected, the extension of short telomeres decreased the frequency of STLs (Table 1).

We also quantified SV40 sequences in hTERT expressing AG11395 cells by qPCR. Unlike WRN expression that caused a slow, gradual reduction in SV40 sequence (Table 2) with increasing PDs, we found a greater initial decrease in SV40 sequence at PD10 with no significant additional change over 80 PDs (Figure 5(c)).

### 3.9. Expression of WRN or Telomerase Did Not Change the Rate of Telomere Sister Chromatid Exchange (T-SCE) in AG11395

To understand the effect of WRN expression on telomere sister chromatid exchange we employed CO-FISH. We did not observe a significant change in the T-SCE in WRN expressing cells when compared to empty vector-transfected AG11395 cells (Table 2). The rate of T-SCE in empty vector-transfected, WRN-expressing and telomerase-expressing AG11395 cells is 10.4%, 11.9%, and 9.4%, respectively.

## 4. Discussion

WS patients exhibit an increased incidence of mesenchymal cancers compared with the general population, but little is known about the genetic pathways perturbed in these tumors because few WS tumor-derived cell lines exist [10]. The increased incidence of mesenchymal cancers in WS patients is intriguing because many human sarcomas maintain telomeres by the ALT pathway [10]. When immortalized G5 mouse *Terc* ^−/−^ *Wrn* ^−/−^  clones are injected subcutaneously into severe combined immunodeficiency mice, aberrant telomere recombination coupled with the strong selective pressure to maintain telomere length in the absence of telomerase results in the activation of ALT tumors [12]. These ALT tumors appeared to be type II, but the possibility of ALT type I tumor formation by WRN-deficient cells cannot be ruled out.

It has been suggested that in *S. cerevisiae* ALT type I is generated by the selection of cells in which successive recombination events have taken place in subtelomeric regions [36]. WRN deficiency can facilitate the sub-telomeric recombination in human cells due to the extensive telomere sequence loss during telomere replication [31] and homologous recombination [37] and possibly by increased aberrant recombination between nonidentical (homeologous) DNA sequences [30, 37]. Survivors of such events may generate ALT that maintains telomeres through a mechanism analogous to the type I survival pathway observed in *sgs1*, telomerase-null yeast. The Werner mutant, SV40-immortalized ALT cell line AG11395 maintains telomeres in a mechanism similar to the type I survival pathway of yeast [20, 21]. This cell line offers a unique system to investigate the role of WRN deficiency and tandem array telomeres in human ALT telomere maintenance.

**Figure 6:**
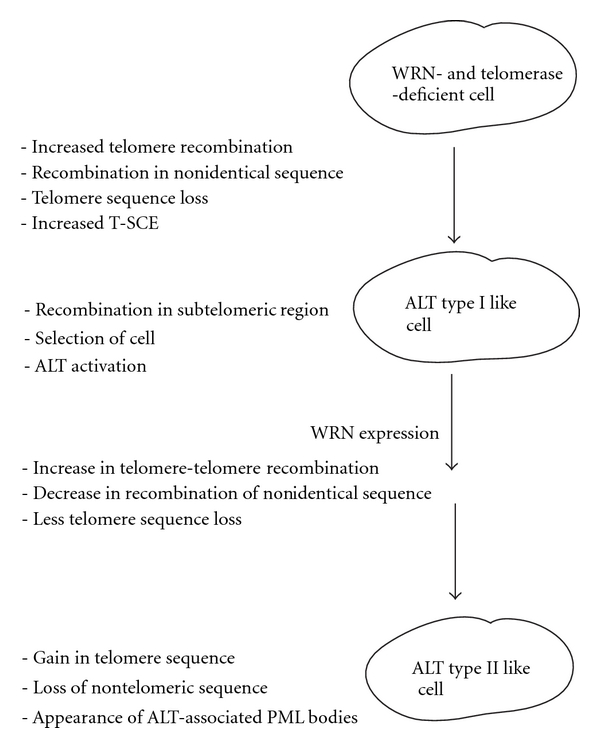
Proposed role of WRN in transformation of ALT type I like telomere to ALT type II like telomere. In the WRN- and telomerase-deficient cell, ALT is activated. Selection of a cell with recombination in subtelomeric region gives rise to ALT type I like telomere. WRN transduction transforms the ALT I like telomere to ALT type II like telomere.

Cohen and Sinclair tested the effect of reintroduction of Sgs1 into type I yeast survivors. This resulted in telomeres with telomere sequence tracts extended by about 300 bp and with decreased numbers of Y′ elements, but it did not fully convert the telomeres to a type II structure [17]. Our results with reintroduction of WRN in AG11395 cells, with increased numbers of telomere sequence repeats and decreased SV40 sequences present at chromosome ends, are consistent with experiments of reintroduction of Sgs1 in yeast Typ1 ALT [17]. These data support the hypothesis that Sgs1 and WRN promote telomere-telomere recombination that results in extension of telomere sequence and reduction of non-telomere sequence, possibly, by decrease in aberrant recombination between nonidentical (homeologous), DNA sequences and telomere sequence loss. The proposed role of WRN in transforming type I ALT like telomeres to type-II-like telomeres are summarized in Figure 6.

Autonomously replicating sequences (*ARSs*) in *Saccharomyces cerevisiae *have been characterized as both origins of DNA replication and as chromatin repressors/silencers. Some (ARSs) that functions in a plasmid are silent as replication origins in their natural chromosomal context. The origin and the silencer activities of *ARS *are modulated by position and chromatin environment. The silencing side of *ARSs *is surfaced in the subtelomere regions, where they enhance and extend the repression signals emitted by the telomere. The effect of sequence elements or chromatin structure on the function of SV40 origin of replication has also been reported [38]. To avoid the sequence dependency, well-characterized plasmids containing the SV40 origin have been extensively used to study SV40 origin function in mammalian cells in the presence of large T antigen.

AG11395 maintains telomeres that have a tandem array structure that contains SV40 sequences, including the SV40 origin of replication, and telomere repeats. To determine if the SV40 origins are functional in AG11395 cells we used a plasmid containing the SV40 origin of replication. We found that unlike WI38 75.1 AG11395 does not support replication of the SV40 origin of replication, even if wild-type T-antigen is provided in trans. The most extensively reported reasons for failure of SV40 origin to support replication of plasmid are cellular inhibitory factors [39] or dominant negative T antigen [40]. For example, WT1 encoded by the Wilms' tumor gene inhibits replication by binding to SV40 origin. Many cellular factors, including the cellular late origin binding protein (LOB) protein (and possibly additional origin-specific proteins), interact with T antigen to prepare the SV40 origin for replication [41]. AG11395 cells may be missing or contain mutated cellular factor(s) required for normal functioning of the SV40 origin of replication.

Alternatively, T antigen functions as an oligomer [42]. Some mutant T antigens can be complemented *in vivo* by providing a wild-type T antigen in trans, while others exhibit a strong trans-dominant-negative phenotype [40]. Although we do not have any direct evidence, but it is possible that the T antigen expressed in AG11395 cells has a dominant negative phenotype for function at the SV40 origin of replication. This would most simply explain the failure to stimulate replication at the SV40 origin in AG11395 when wild-type T antigen was expressed. 

The role of nonfunctional Y′ elements to yeast type I survivors and the role of the SV40 origin of replication in AG11395 telomeres remain unclear. They extend the net length of telomere with interspersed nontelomeric sequences and may serve as chromatin repressors like the ARS in Y′ element of type I ALT telomere in yeast.

A small number of AG11395 cells express APBs after WRN expression. Endogenous WRN colocalizes with PML protein in ALT cells [13]. However, there is no simple correlation between ALT activity level and the number of APBs or APB-positive cells. The proportion of APB-positive cells can be greatly increased by methionine starvation, by DNA-damaging agents, or by restoring p53 function in ALT cells [43, 44]. In each of these cases, the treatments that induced APBs also caused growth arrest. An ectopically expressed GFP-WRN partially colocalized in large nucleoplasmic foci with the endogenous PML protein in UV-irradiated U-2OS cells [45]. PML shell formation starts from the site of PML-WRN contact and proceeds until the PML layer is formed on entire surface of a WRN body [46].

At present it is not clear how WRN facilitates APB formation. One possibility is that WRN is modulating an arrest phenotype. For example, Werner syndrome cells escape hydrogen-peroxide-induced cell proliferation arrest [47]. We anticipate that, given the expression of T antigen, AG11395 cells do not arrest in G1 phase of cell cycle. However, it is possible that restoring WRN facilitates the arrest of few cells accompanied by the induction of APBs in G2/M phase following spontaneous oxidative damage.

The expression of telomerase in AG11395 resulted in a rapid expansion of telomere sequences and in a decrease in the frequency of STLs. Although the expression levels of WRN protein in the clones that were selected for analysis varied significantly, we did not observe any significant correlation in STL or telomere sequence gain with the level of WRN expression. We observed rapid loss of SV40 sequences by PD 10 that persisted, but did not increase, over 80 PDs. As ALT is a recombination-based mechanism, the rapid loss of SV40 sequences is consistent with increased deletions occurring during homologous recombination in the absence of WRN [37]. That no further loss of SV40 sequences was observed after 10 PDs in the presence of increased telomere repeats is consistent with the presence of telomere repeat tracts protecting the residual SV40 repeats and is in accord with the more recent observation that telomerase acts at the majority of telomeres in the cell at each cell division [48]. If telomerase only acted on the shortest telomeres in the cell, a continued decline in the presence of SV40 sequences at telomeres would be expected as the longer SV40 containing telomeres over time reach the “critical” length and are subsequently extended by telomerase.

## 5. Conclusion

Our results indicate that the Werner protein plays a fundamental role in determining the telomere maintenance mechanisms in transformed cells. In the human WRN mutant cell line AG11395, exogenous expression of Werner protein facilitates the transition of ALT type I like telomeres to type II like telomeres. The increased incidence of mesenchymal cancers observed in WS patients may relate to the nature of the telomere maintenance program activated in these tumors. Besides giving valuable diagnostic and prognostic information, telomere maintenance mechanisms are a potential therapeutic target for cancer treatment. The status of Werner protein expression in tumor cells may be helpful in designing therapeutic strategies.

## Figures and Tables

**Table 1:** WRN and telomerase expression decreases sister telomere loss (STL) in AG11395. Fifteen metaphases per cell line were analyzed. *P* values indicated are from the Wilcoxon two-sided ranks test, comparing the WRN and telomerase-expressing clones to the pooled empty vector-infected controls.

Cell line	STL/cell	Significance
AG11395 EV1	11.4	
AG11395 EV2	9.2	
AG11395 EV3	10.6	
AG11395 WRN1	7.1	*P* < 0.0005
AG11395 WRN2	6.9	*P* < 0.01
AG11395 WRN3	4.4	*P* < 0.0001
AG11395 hTERT	2.8	*P* < 0.00001

**Table 2:** Effects of WRN and telomerase expression in AGO11395 cells.

	Empty vector	WRN expressing	Telomerase expressing
	AGO11395	AGO11395	AGO11395
STL/cell	9.2–11.4	4.4–7.1	2.8
SV40 origin sequence loss	—	20–25%	40–50%
Telomere length in AFUs	449−577	771–919	872
APBs	not present	present	not present
T-SCE	10.4%	11.9%	9.4%

## References

[1] Hayflick L, Moorhead PS (1961). The serial cultivation of human diploid cell strains. *Experimental Cell Research*.

[2] Counter CM, Avilion AA, Lefeuvre CE (1992). Telomere shortening associated with chromosome instability is arrested in immortal cells which express telomerase activity. *The EMBO Journal*.

[3] Henson JD, Neumann AA, Yeager TR, Reddel RR (2002). Alternative lengthening of telomeres in mammalian cells. *Oncogene*.

[4] Siddiqa A, Cavazos DA, Marciniak RA (2006). Targeting telomerase. *Rejuvenation Research*.

[5] Villa R, Daidone MG, Motta R (2008). Multiple mechanisms of telomere maintenance exist and differentially affect clinical outcome in diffuse malignant peritoneal mesothelioma. *Clinical Cancer Research*.

[6] Zimmermann S, Martens UM (2007). Telomeres and telomerase as targets for cancer therapy. *Cellular and Molecular Life Sciences*.

[7] Multani AS, Chang S (2007). WRN at telomeres: implications for aging and cancer. *Journal of Cell Science*.

[8] Opresko PL, Calvo JP, von Kobbe C (2007). Role for the Werner syndrome protein in the promotion of tumor cell growth. *Mechanisms of Ageing and Development*.

[9] Agrelo R, Cheng WH, Setien F (2006). Epigenetic inactivation of the premature aging Werner syndrome gene in human cancer. *Proceedings of the National Academy of Sciences of the United States of America*.

[10] Ozgenc A, Loeb LA (2006). Werner Syndrome, aging and cancer. *Genome Dynamics*.

[11] Henson JD, Hannay JA, McCarthy SW (2005). A robust assay for alternative lengthening of telomeres in tumors shows the significance of alternative lengthening of telomeres in sarcomas and astrocytomas. *Clinical Cancer Research*.

[12] Laud PR, Multani AS, Bailey SM (2005). Elevated telomere-telomere recombination in WRN-deficient, telomere dysfunctional cells promotes escape from senescence and engagement of the ALT pathway. *Genes and Development*.

[13] Johnson FB, Marciniak RA, McVey M, Stewart SA, Hahn WC, Guarente L (2001). The *Saccharomyces cerevisiae* WRN homolog Sgs1p participates in telomere maintenance in cells lacking telomerase. *The EMBO Journal*.

[14] Lundblad V, Szostak JW (1989). A mutant with a defect in telomere elongation leads to senescence in yeast. *Cell*.

[15] Lundblad V, Blackburn EH (1993). An alternative pathway for yeast telomere maintenance rescues est1- senescence. *Cell*.

[16] Teng SC, Zakian VA (1999). Telomere-telomere recombination is an efficient bypass pathway for telomere maintenance in *Saccharomyces cerevisiae*. *Molecular and Cellular Biology*.

[17] Cohen H, Sinclair DA (2001). Recombination-mediated lengthening of terminal telomeric repeats requires the Sgs1 DNA helicase. *Proceedings of the National Academy of Sciences of the United States of America*.

[18] Lee JY, Mogen JL, Chavez A, Johnson FB (2008). Sgs1 RecQ helicase inhibits survival of *Saccharomyces cerevisiae* cells lacking telomerase and homologous recombination. *Journal of Biological Chemistry*.

[19] Yeager TR, Neumann AA, Englezou A, Huschtscha LI, Noble JR, Reddel RR (1999). Telomerase-negative immortalized human cells contain a novel type of promyelocytic leukemia (PML) body. *Cancer Research*.

[20] Fasching CL, Bower K, Reddel RR (2005). Telomerase-independent telomere length maintenance in the absence of alternative lengthening of telomeres-associated promyelocytic leukemia bodies. *Cancer Research*.

[21] Marciniak RA, Cavazos D, Montellano R, Chen Q, Guarente L, Johnson FB (2005). A novel telomere structure in a human alternative lengthening of telomeres cell line. *Cancer Research*.

[22] Saito H, Moses RE (1991). Immortalization of Werner syndrome and progeria fibroblasts. *Experimental Cell Research*.

[23] Kurien BT, Scofield RH (2006). Western blotting. *Methods*.

[24] Marciniak RA, Lombard DB, Johnson FB, Guarente L (1998). Nucleolar localization of the Werner Syndrome protein in human cells. *Proceedings of the National Academy of Sciences of the United States of America*.

[25] Poon SS, Lansdorp PM (2001). Quantitative fluorescence in situ hybridization (Q-FISH). *Current Protocols in cell Biology*.

[26] Bailey SM, Goodwin EH, Cornforth MN (2004). Strand-specific fluorescence in situ hybridization: the CO-FISH family. *Cytogenetic and Genome Research*.

[27] Zakian VA (1996). Structure, function, and replication of *Saccharomyces cerevisiae* telomeres. *Annual Review of Genetics*.

[28] Rehman MA, Fourel G, Mathews A (2006). Differential requirement of DNA replication factors for subtelomeric ARS consensus sequence protosilencers in *Saccharomyces cerevisiae*. *Genetics*.

[29] Rehman MA, Yankulov K (2009). The dual role of autonomously replicating sequences as origins of replication and as silencers. *Current Genetics*.

[30] Myung K, Datta A, Chen C, Kolodner RD (2001). SGS1, the *Saccharomyces cerevisiae* homologue of BLM and WRN, suppresses genome instability and homeologous recombination. *Nature Genetics*.

[31] Crabbe L, Verdun RE, Haggblom CI, Karlseder J (2004). Defective telomere lagging strand synthesis in cells lacking WRN helicase activity. *Science*.

[32] Grobelny JV, Godwin AK, Broccoli D (2000). ALT-associated PML bodies are present in viable cells and are enriched in cells in the G_2_/M phase of the cell cycle. *Journal of Cell Science*.

[33] Perrem K, Colgin LM, Neumann AA, Yeager TR, Reddel RR (2001). Coexistence of alternative lengthening of telomeres and telomerase in hTERT-transfected GM847 cells. *Molecular and Cellular Biology*.

[34] Counter CM, Hahn WC, Wei W (1998). Dissociation among in vitro telomerase activity, telomere maintenance, and cellular immortalization. *Proceedings of the National Academy of Sciences of the United States of America*.

[35] Counter CM, Meyerson M, Eaton EN (1998). Telomerase activity is restored in human cells by ectopic expression of hTERT (hEST2), the catalytic subunit of telomerase. *Oncogene*.

[36] Makovets S, Williams TL, Blackburn EH (2008). The telotype defines the telomere state in *Saccharomyces cerevisiae* and is inherited as a dominant non-Mendelian characteristic in cells lacking telomerase. *Genetics*.

[37] Yamamoto ML, Reliene R, Oshima J, Schiestl RH (2008). Effects of human Werner helicase on intrachromosomal homologous recombination mediated DNA deletions in mice. *Mutation Research*.

[38] Chen PH, Tseng WB, Chu Y, Hsu MT (2000). Interference of the simian virus 40 origin of replication by the cytomegalovirus immediate early gene enhancer: evidence for competition of active regulatory chromatin conformation in a single domain. *Molecular and Cellular Biology*.

[39] Anant S, Axenovich SA, Madden SL, Rauscher FJ, Subramanian KN (1994). Novel replication inhibitory function of the developmental regulator/transcription repressor protein WT1 encoded by the Wilms’ tumor gene. *Oncogene*.

[40] Castellino AM, Cantalupo P, Marks IM, Vartikar JV, Peden KWC, Pipas JM (1997). Trans-dominant and non-trans-dominant mutant simian virus 40 large T antigens show distinct responses to ATP. *Journal of Virology*.

[41] Baur CP, Klausing K, Scheffner M, Stahl H, Knippers R (1988). Protein-DNA interactions at the Simian Virus 40 origin of replication. *Biochimica et Biophysica Acta*.

[42] San Martin MC, Gruss C, Carazo JM (1997). Six molecules of SV40 large T antigen assemble in a propeller-shaped particle around a channel. *Journal of Molecular Biology*.

[43] Fasching CL, Neumann AA, Muntoni A, Yeager TR, Reddel RR (2007). DNA damage induces alternative lengthening of telomeres (ALT)-associated promyelocytic leukemia bodies that preferentially associate with linear telomeric DNA. *Cancer Research*.

[44] Jiang WQ, Zhong ZH, Nguyen A (2009). Induction of alternative lengthening of telomeres-associated PML bodies by p53/p21 requires HP1 proteins. *Journal of Cell Biology*.

[45] Blandert G, Zalle N, Daniely Y, Taplick J, Gray MD, Oren M (2002). DNA damage-induced translocation of the Werner helicase is regulated by acetylation. *Journal of Biological Chemistry*.

[46] Vaitiekunaite R, Butkiewicz D, Krześniak M (2007). Expression and localization of Werner syndrome protein is modulated by SIRT1 and PML. *Mechanisms of Ageing and Development*.

[47] Von Kobbe C, May A, Grandori C, Bohr VA (2004). Werner syndrome cells escape hydrogen peroxide-induced cell proliferation arrest. *The FASEB Journal*.

[48] Zhao Y, Sfeir AJ, Zou Y (2009). Telomere extension occurs at most chromosome ends and is uncoupled from fill-in in human cancer cells. *Cell*.

